# Randomised trial on effect of involving media reporters in salt reduction programme to increase media reports and the public’s knowledge, belief and behaviors on salt and health: Changzhi reporters trial

**DOI:** 10.1371/journal.pone.0252989

**Published:** 2021-07-20

**Authors:** Zhifang Li, Xiangxian Feng, Tao Wu, Lijing Yan, Paul Elliott, Yangfeng Wu

**Affiliations:** 1 Department of Public Health and Preventive Medicine, Changzhi Medical College, Changzhi, Shanxi, China; 2 Epidemiology, Janssen Research and Development, Beijing, China; 3 Global Health Research Center, Duke Kunshan University, Kunshan, Jiangsu, China; 4 MRC Centre for Environment and Health, School of Public Health, Imperial College London, London, United Kingdom; 5 National Institute for Health Research Biomedical Research Centre, Imperial College London, London, United Kingdom; 6 Peking University Clinical Research Institute, Peking University First Hospital, Beijing, China; 7 Peking University School of Public Health, Beijing, China; 8 The George Institute for Global Health at Peking University Health Science Center, Beijing, China; State University of Rio de Janeiro, BRAZIL

## Abstract

**Objective:**

To assess the effects of a novel mass media intervention in increasing media reports on salt and health by involving media reporters in a scientifically well designed salt reduction trial.

**Methods:**

We recruited and trained 66 media reporters in Changzhi, Shanxi province, China to conduct a randomized controlled trial on blood pressure lowering effect of salt substitute in Dec, 2012 and Jan 2013 among their own relatives or friends (253 from 129 families in the salt substitute arm and 263 from 133 families in the control arm for two months). We shared trial results and other information on salt and health with the reporters within a month after the trial. We monitored all local newspapers for the number of relevant articles in 3 months before, 3 months during and 3 months after the intervention and at the 6^th^, 12^th^, 18^th^, 24^th^ and 48^th^ months after the intervention. Additionally, we conducted two independent surveys on knowledge, belief and behaviours of salt and health among local citizens before and after the intervention.

**Results:**

As expected, systolic blood pressure was reduced significantly more in the salt substitute than the control group (-4.7±11.0 mmHg vs -2.6±10.3 mmHg, p<0.001) in the randomized trial. The monthly mean number of relevant articles increased from 0.7 before to 1.7 during (p = 0.263), and further to 6.0 after the intervention (p<0.001), and varied from 2 (p = 0.170) to 4 (p = 0.008) from the 6^th^ to 48^th^ month; the awareness of knowledge on salt and health among local citizens improved significantly after the intervention.

**Conclusions:**

Media reporters’ participation in a well-designed salt reduction trial significantly increased the number of relevant media reports, and the effect was sustained for a prolonged period. Future mass media public health education programs should consider this innovative strategy for better and sustained impacts.

## Introduction

In China, the prevalence of hypertension has been increasing. The most recent official prevalence of hypertension in 2012–15 was reported to be 25.2% in adults, translating to over 200 million patients with high blood pressure, making China a country with the highest number of patients with hypertension in the world [1]. Hypertension-related diseases such as stroke and coronary heart disease as well as deaths from these diseases have also been increasing [2,3].

Population salt reduction has been identified by the World Health Organization (WHO) as one of the three ‘best buys’ for prevention and control of cardiovascular disease globally [4]. Many observational studies [5–10], animal studies [11,12] and randomized clinical trials [13,14] have confirmed that reducing salt intake can reduce blood pressure levels [14,15], thereby reducing the incidence of cardiovascular diseases [2,16–20]; recent observational studies showing a purported ‘U’ shape curve between salt intake and cardiovascular mortality [21,22] have been criticized on methodological grounds [23,24].

WHO and professional societies around the world have recommended lower salt intake for prevention and control of hypertension [25]. Salt reduction has become a global action in prevention and control of hypertension and cardiovascular disease. However, significant challenges remain to implement such a policy widely. Per capita salt intake is 8–13 g per day in some European countries, such as 9.5 g per day in the UK, 12 g per day in China, all markedly higher than the WHO recommended 5 g per day [26].

Among strategies for population salt reduction, leveraging the media to educate the public to reduce salt intake and promote health could be convenient and cost-effective, especially in countries like China where most of salt intake derives from salt added during cooking at home [27]. Mass media plays a critical role in public health promotion and disease prevention [28–30]. However, current methods of engaging mass media for public health education either have little effect or are limited to having a one-off or transitory effect that does not last long [31–33].

In this study, we described an innovative approach, involving media reporters to implement a trial designed to rigorously test the effect of salt substitute in lowering blood pressure among their own families, neighbours or relatives, which aims to increase the number of media reports on salt and health in the local media over short and long-term; and therefore to further change the population’s knowledge, belief and behaviours on salt intake and health among local citizens.

## Materials and methods

### Study design

**Fig 1:**
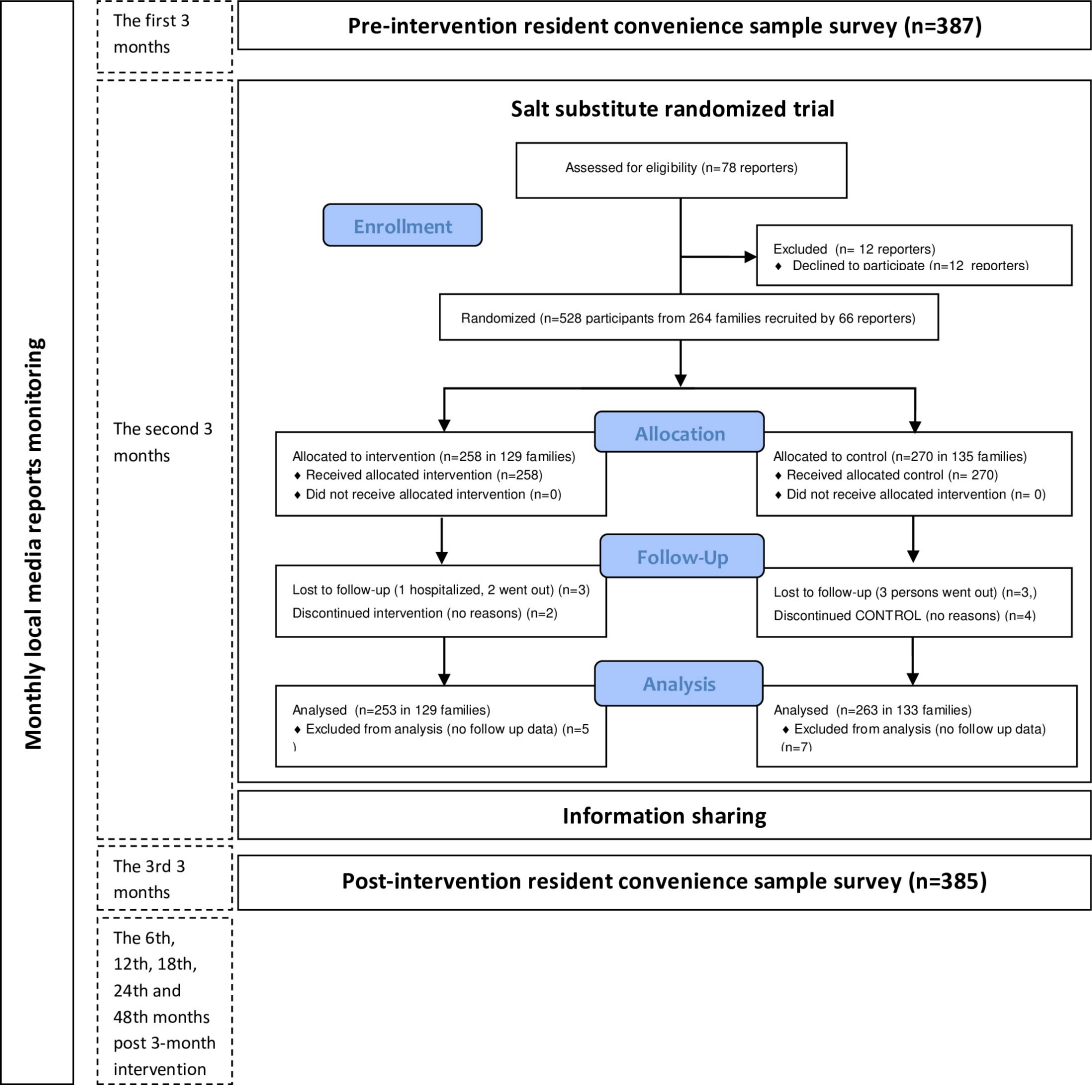
Study design and patient flow chart.

We employed a before-and-after comparative study design to evaluate the effects of the intervention. The study was carried out in Changzhi, Shanxi Province of China, which is a medium size city with a total population of 3.46 million. The intervention targeted all local newspaper reporters and included a reporters-implemented salt reduction randomized trial followed by dissemination of trial results and additional information sharing. The intervention took place for 3 months in total from Dec, 2012 to Feb, 2013, two months for the trial and one month for information sharing. We monitored all local newspapers for the number of relevant articles in 3 months before, 3 months during and 3 months after the intervention and at the 6th, 12th, 18th, 24th and 48th months after the 3-month intervention. In addition, we conducted two independent surveys among local residents to understand the changes in knowledge, belief and behaviour before and after the intervention (Fig 1). The work of monitoring newspapers ended in Feb, 2017.

The study was approved by the Peking University Institutional Review Board, Beijing, China (#IRB00001052-12064). All participants provided written informed consent. The study design information was submitted to the ClinicalTrials.gov (NCT03074851) prospectively but shown retrospectively, due to the mistake we made by our own inexperience with the system then. The mistake delayed releasing the submitted information until it was found in 2017.

### The intervention

#### 1) Randomized controlled salt substitute trial implemented by reporters

The main part of the study intervention was designed as a randomized controlled salt substitute trial to be implemented by local newspaper reporters. To be eligible, the reporters had to have a national press reporter certificate with health column reporters preferred, had planned to live in Changzhi in the next 12 months, had easy access to at least 4 families including relatives, friends or neighbors who would be asked to participate in the study. Among a total of 78 local media reporters, 12 refused to take part, leaving 66 reporters participated in the study.

We chose salt substitute as the intervention for the randomized trial because its effect in lowering blood pressure had previously been demonstrated in Chinese populations [34–38]. The trial was planned for a period of two months and each study reporter was responsible to recruit 4 families to participate in the trial, according to the following inclusion and exclusion criteria: had at least two family members aged 50 years or more, had no plan to move within 6 months, and living conveniently for the reporter in charge to take the blood pressure measurements in their leisure time. For an individual to be eligible, he/she should be at least 50 years old and consume 3 meals a day at home for at least 5 days a week. We excluded those with severe kidney disease or receiving potassium-sparing diuretics. The 66 study reporters recruited a total of 262 families and 516 study participants. All study participants provided written informed consent.

The families were randomly divided into two groups: intervention group and control group in a 1:1 ratio. The randomization stratified by the reporters was done by an independent staff at the Changzhi Medical College using the random digit table. The family allocated to the intervention group received free salt substitute (70% sodium chloride and 30% potassium chloride). Sufficient salt substitute was provided to the entire family for the use of two months. Families in the control group received no salt substitute and continued to use the regular salt. We chose not to use a salt enriched placebo for the control group for the following considerations: 1) We were interested in observing the effect of the trial in changing the media reporters’ will and writing to report on salt intake and health, rather than replicate the findings of the previous studies; 2) Blinding would increase the difficulty and cost of implementing the trial, and our budge was constrained; 3) Salt is a daily necessity, and the participants would have to use regular salt anyway if they were randomized into the control group; 4) When the study was done, the salt substitute had not been marketed and no salt reduction campaigns took place in the study area, both the study participants and media reporters were not familiar with the product and its function in lowering blood pressure.

Before and after the intervention, the reporters measured the blood pressure for all study participants in both intervention and control groups at the participants’ homes using a validated automatic blood pressure monitor (Omron HEM-7301-IT) with an appropriately sized cuff. After participants had rested for 5 minutes in a quiet room, two readings were taken in the right arm at 1-minute interval with the participants in the sitting position and the arm supported at heart level. We used the average of the two measurements in all analyses. Body weight and height were measured in participants without shoes or heavy clothes, following a standardized protocol. The reporters sent the measurement results via mobile short messages to the data center at Changzhi Medical College. All reporters received training by the study staff before the implementation of the study. A project manager was recruited to manage the trial and remind reporters of on-time blood pressure measurements.

#### 2) Sharing information on salt and health with reporters

We analyzed the results of the built-in trial with the principle of intention to treat. Study results were shared with the reporters through emails. In addition, text messages with information on salt and health were sent by the study personnel, one specific topic each day, to all participating reporters in the month following the two-month salt substitute trial.

### Evaluation of the intervention

#### 1) Media reports monitoring

Personnel who were independent of the study were recruited, to collect relevant articles published in the local newspapers in the 3 months before intervention (September to November, 2012), 3 months during intervention (December 2012 to February 2013), 3 months after intervention (March to May 2013), and also at the 6^th^、12^th^、24^th^、48^th^ months after the intervention (The initial plan was to follow up to the 12 months after the intervention. We decided to extend to 48 months after the intervention to better understand the longer term effect). The relevant articles were defined as any type of article that contained description of ‘salt’, ‘high salt intake’, ‘salt reduction’, ‘salt substitute’, or ‘types of salt’ in relation to prevention and control of chronic diseases. On a weekly basis, the designated personnel went through all newspaper articles to record all relevant articles meeting the inclusion criteria. Before the data collection, the personnel was trained by the first author of the study on the methods and criteria to be used.

#### 2) Surveys of local residents

At the weekend immediately before and the weekend immediately after the 3-months intervention (2-months salt substitute intervention, followed by 1-month information sharing), we conducted two independent surveys of local residents in the same places, the largest shopping center and the largest public square in the city. We adopted the convenience sampling method to intercept volunteer residents according to a pre-defined number of people in each age- and sex-group. In each survey, we aimed to survey 100 individuals in each 10-year age group, starting from age 31–40 years to age 51–60 years, with 50 individuals in the group age 61 years and above. In each age group, we aimed for half men and half women. In total, 772 men and women were surveyed, 387 in the survey before and 385 in the survey after the intervention.

We used self-developed questionnaires for the interview of the residents, and only five simple questions about salt and health were asked in addition to age, sex, occupation, education and place of residence. The multiple-choices questions included 1) “Do you believe that high salt intake is harmful to your health? (Choices: yes, no, and don’t know)”; and 2) “if yes, what diseases could it lead to or aggravate? 3) “Do you know how much salt a day the Chinese Nutrition Society recommend to take in? (Choices: 3 g/d, 6 g/d, 10 g/d, and don’t know)”; 4) “What type of salt do you use at home (choices: iodine-fortified, Se-fortified, salt substitute, regular salt, and don’t know), and 5) “Do you know how much salt you take every day? and if yes, how much?” We used answers to question #1 to count the number who believe salt is harmful to health, answers to questions #2 and 3 for the number who were aware of sodium and health issues, and answers to question #4 for the number who complied with lower sodium choices. We derived a new variable of ‘paying attention to the amount of salt intake’ (as a behaviour) from the question #5 by accounting for those who answered ‘yes’ to the question and were able to give the amount specifically.

### Sample size estimation

For the built-in salt substitute trial by reporters, we estimated the sample size on the basis of our previous randomized trials [33,34,39], which indicated an effect size of the salt substitute of between 5.4 mmHg to 9.1 mmHg for systolic blood pressure (SBP) with a standard deviation about 13.4 mmHg to 22.3 mmHg. We assumed that two-sided α = 0.05, intra-family correlation coefficient = 0.4 and % lost to follow up = 10%. A total of 240 families (480 participants) with 1:1 ratio of intervention to control had 85% power to detect an effect size of 5 mmHg in SBP with standard deviation of the changes in SBP = 15 mmHg.

For the series of media reports monitoring study, all local newspapers were monitored. We assumed that the baseline rate would be less than one article per newspaper per month, and an increase to five articles per month would have 80% power to be detected at 5% significance level.

For the resident convenience sample surveys, with reference to previous studies [37], we assumed that the proportion of Chinese residents with the knowledge, belief and behaviour on salt intake and health in this part of China was 30%, 60% and 30% respectively. We assumed α = 0.05, a study sample size with 400 individuals in each survey would have 85% power to detect an effect size of 10% change in each of these proportions.

### Statistical analysis

The difference in number of relevant articles and proportions of knowledge, belief and behaviour between pre- and post-intervention was compared using the Chi-square test. The changes in systolic and diastolic blood pressure between intervention and control in the reporters-implemented randomized trial were compared using generalized estimation equation model adjusting for clustering (families) effect and baseline blood pressure levels. All statistical tests were two-sided and the level of significance was set at P = 0.05. The statistical software package SPSS 20.0 was used for all data analysis.

## Results

### The characteristics of study reporters

Out of the 66 reporters participating in the study, 64% were women, 79% had college or above education, 50% were reporting on news and another 17% on health and education (Table 1).

**Table 1:** Demographic characteristics of reporters (n = 66) participating in the study.

Factors	Category	N	%
**Gender**	Male	24	36.4
	Female	42	63.6
**Age**	<30 yrs	9	13.6
	30–39 yrs	21	31.8
	40–49 yrs	19	28.8
	> = 50 yrs	17	25.8
**Education**	Less than college	14	21.2
	College and above	52	78.8
**Report area**	News	33	50.0
	Health and Education	11	16.7
	Others (Economy, Entertainment, Sports, etc.)	22	33.3
**Number of family members**	1–2	75	28.6
	3–4	145	55.3
	5 and above	42	16.0

### Results from the reporters-implemented randomized trial

A total of 516 participants from 262 families were recruited for the randomized trial from Nov 26, 2012 to December 6, 2012. Among them, 253 from 129 families in the intervention arm and 263 from 133 families in the control arm completed the trial. The two groups were highly comparable in terms of age and sex distribution at baseline (Table 2).

**Table 2:** Characteristics of the study population and the pre-and-post-intervention changes in systolic (SBP) and diastolic blood pressure (DBP) in the randomized salt substitute trial, by intervention group.

Variables	Intervention	Control	P values
**Population characteristics**			
** Number of families**	129	133	
** Number of participants**	253	263	
** Mean age (yrs±SD)**	59.3±8.7	59.2±8.7	0.869
** Women (%)**	51.0	49.0	0.598
**Baseline blood pressure (mmHg)**			
** SBP**	128.9±13.5	126.8±13.5	0.083
** DBP**	82.0±10.1	81.3±11.7	0.482
**Change in blood pressure from baseline (mmHg)**			
** SBP**	-4.7±11.0	-2.6±10.3	<0.001
** DBP**	-2.4±7.9	-1.1±8.5	<0.001

After 2 months of intervention, SBP in both groups fell but in the intervention group declined significantly more. DBP showed the same trend (Table 2). After adjusting for baseline blood pressure and clustering effect, it showed that SBP and DBP in intervention group declined significantly more than that in control group. There was no adverse event reported during the trial in either group.

### Results from the media monitoring

**Fig 2:**
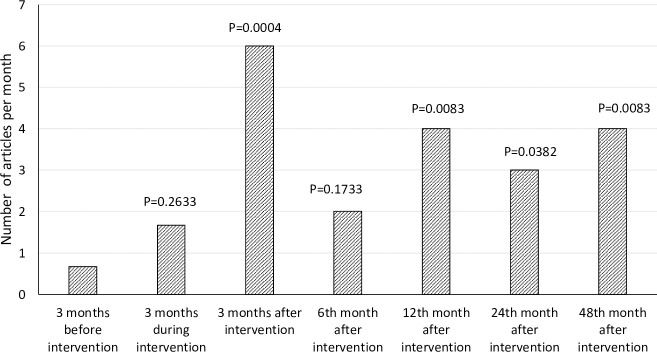
Average number of relevant articles before, during and after the interventions in Changzhi, China.

The number of relevant articles in local media was 0.7 per month before the intervention and it increased to 1.7 per month (p = 0.263) during the intervention and to 6 per month (p<0.001) immediately after the intervention. It remained at about 2 (p = 0.173) to 4 (p = 0.008) articles per month, which was several folds the number published before the intervention (Fig 2).

### Results from the local residents’ surveys

**Fig 3:**
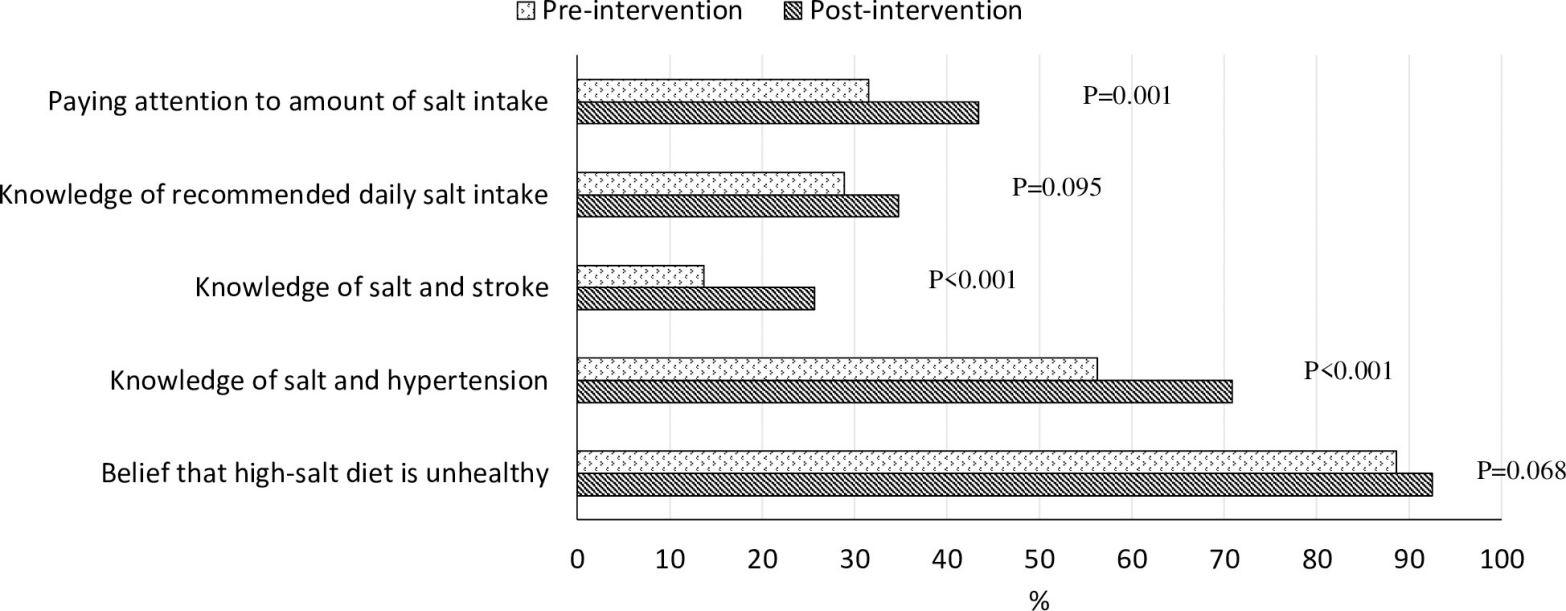
Changes in knowledge, belief and behaviour before and after intervention.

Table 3 compares the demographic characteristics of the residents who participated in the convenience sample surveys before and after the intervention; no major differences were seen in terms of age, gender, occupation, highest education, and sources of health information in the two samples. Compared with the pre-intervention survey, the proportion of residents who were aware of knowledge on salt and health (56.3% vs 70.9% on hypertension; 13.7% vs 25.7% on stroke) was significantly higher in the post-intervention sample (Fig 3). In addition, the proportion of residents who paid attention to amount of salt intake increased significantly from 31.5% to 43.4%. However, the proportion of residents who were aware of the recommended amount of salt intake (28.9% vs 34.8%) and who believed in the harm of high salt intake (88.6% vs 92.5%) were not statistically significant between the two samples.

**Table 3:** Demographic characteristics of study participants in the resident convenience sample surveys before and after the intervention.

Factors	Category	Pre-intervention (n = 387)	Post-intervention (n = 385)	P 值
**Women**		49.9	51.7	0.614
**Age**	30~40 yrs	27.4	28.8	0.710
	41~50 yrs	27.9	28.8	
	51~60 yrs	29.2	25.5	
	>60 yrs	15.5	16.9	
**Occupation**	Worker^a^	25.8	28.3	0.294
	Farmer^b^	19.1	19.2	
	Administrative staff ^c^	13.7	13.8	
	Self-employed^d^	22.5	25.5	
	Unemployed^e^	18.9	13.3	
**Education**	Never attended school	7.5	5.2	0.195
	Primary school	16.5	13.3	
	Junior middle school	40.3	38.4	
	High school and equitable	25.1	31.2	
	College and above	10.6	12.0	

^a^Including people who work in State-owned or Certain scale private enterprise with a stable income.

^b^ Including people mainly engaged in agricultural work.

^c^ Including people who work in state-run institutions with a stable income, such as doctors, teachers etc.

^d^Icluding people without fixed work and fixed income, such as waiters, cleaners etc.

^e^Including people who are unemployed and without any income, such as housewives, etc.

## Discussion

Our study demonstrated that inviting media reporters to participate in a rigorously designed intervention trial, asking them to be responsible for study participant recruitment, intervention delivery and outcome measurement, and having the trial results shared with them, could significantly increase the number of media reports of relevant articles–in this case, by 3 to 5-fold,with an effect that lasted over the long term (up to 4 years) (See Fig 2). The effect of intervention was not limited to an increase in the number of relevant articles, but was also observed in city residents’ knowledge and behaviour relevant to salt intake and health (See Fig 3). The study offers an effective and innovative approach to mass media health education and involvement that could be taken and extended to other areas of health interest.

Previous studies have found that mass media often has little effect on people’s behaviour, particularly for on-going behaviours such as diet, physical activity and smoking; while short-term changes can be achieved, sustained effects are difficult to maintain after the campaigns end [31]. In the present study, how were we able to achieve an increasing number of media reports on salt and health and maintain the effect for years? According to the Theory of Reasoned Action [32] the reporters’ adoption of writing on salt and health is a function of their intention to do it. The intention to write on the topic is, in turn, a function of their attitude toward writing on that topic and of perceived social norms and motivations to comply. Reporters, especially in the health and science fields, tend to be well-educated with good scientific reasoning. If they had the chance to do a study by themselves and the study used a design they trust to generate a solid conclusion, they would believe in that conclusion. The randomized controlled trial is such a robust study design. Compared with being presented with results from studies done by others, often a stranger, receiving results from their ‘own study’ in which they are the actual ‘players’ may positively affect their beliefs in what is concluded from the study and increase their motivation to report on the topic.

The evaluation of mass media campaigns is often difficult. Randomized controlled trials, although being considered the ‘gold standard’, are often not feasible or appropriate for the evaluation of mass media campaigns [32]. In this study, we used a traditional method of newspaper article review to monitor the number of relevant articles published in local newspapers, periodically for over 4 and half years. The clear relationship between our intervention and the change in number of relevant articles subsequently published supports a potentially causal association between the two. In addition, we used the same method in conducting two convenience sample surveys, one before and one after the intervention, and we chose the same places to recruit the survey interviewees–both the biggest mall and the most popular public square–in order to ensure the compatibility between the two samples.

## Limitations

Our study has several limitations. First, we only invited newspaper reporters. Other media reporters including those from TV and social media were not included. Thus, the effect of our intervention on the public’s knowledge, belief and behaviours may have been under-estimated compared with what might be achieved. It explained why the intervention effect on KABs was not as large as expected, i.e. people paying attention to amount of salt intake still below 50% after the intervention. Second, it is possible that the increase in numbers of relevant articles published and public knowledge and behaviours resulted from other interventions and dissemination on the importance of salt on health beyond the local trial. While this possibility cannot be ruled out, we are unaware of any national or regional campaigns that would have resulted in such a rapid change in media interest on this particular topic. Third, we can not entirely eliminate the possibility that a reporter who had biased view on salt substitute would introduce a biased intervention effect on blood pressure in the build-in randomized salt substitute trial, due to its open nature. However, we believe that possibility should be very minor if existed. The reasons are: 1) The information sharing undertook after the randomized trial; 2) Blood pressure was measured using an auto-device; 3) Most of the media reporters in China are not having a medical degree. Their level of knowledge on salt and health were almost the same as that of the common people in the society; 4) Salt substitute was not available on the local market then and the reporters were unfamiliar with it too; 5) The number of media reports did not increase during the randomized trial implementation and increased sharply right after the trial, indicating most of the reporters were waiting for the results from the trial before they took any action. Fourth, we did not measured blood pressure in the KAB surveys and could not measure the change in population blood pressure level. However, since the two cross-sectional surveys took part in only 3 months and the change in climate and air temperature might affect blood pressure significantly, the usefulness of measuring blood pressure changes in such a short period should be limited. Last, we did not plan and conduct more surveys after the intervention due to financial constraints. Hence, the long term effects of the intervention on population knowledge, belief and behaviour remain unknown.

## Conclusion

The study demonstrated that involving media reporters in conducting a rigorously designed salt reduction trial significantly increased the number of relevant media articles, and the effect was sustained for an extended period. Such a strategy is novel and we used several methods and outcome measures to evaluate the impact of this strategy. If substantiated by studies in other locations, we recommend mass media public health education programmes consider adopting a similar strategy for more effective and sustained impacts than traditional approaches.

## Supporting information

S1 Checklist CONSORT 2010 checklist of information to include when reporting a randomised trial*. (DOC)

S1 File Dataset for the convenience sample surveys. (XLSX)

S2 File Dataset for the randomized salt substitute trial. (XLSX)

S3 File Study protocol in English. (PDF)

S4 File Study protocol in Chinese. (PDF)
