# Supplementary material for: Randomised trial on effect of involving media reporters in salt reduction programme to increase media reports and the public’s knowledge, belief and behaviors on salt and health: Changzhi reporters trial
Source: PLoS One. 2021 Jul 20;16(7):e0252989. doi: 10.1371/journal.pone.0252989 (PMC8294101; doi:10.1371/journal.pone.0252989)
Supplement: S3 File — (PDF) [file pone.0252989.s004.pdf]

Effect of journalists involving scientific study on news report

Participating institutions and universities

Chinese institutions	Foreign institutions
Peking university Health Science center<br>Changzhi Medical College	Imperial College London

Principal members of the Study Group

Project Manager: Yangfeng Wu. Clinical Institute of Medical Sciences, Peking University

Project Coordinator： Zhifang Li. Changzhi Medical College Foreign Project

Manager： Paul Elliott. Imperial College, London

Abstract

Objective:

To assess the effects of a novel mass media intervention in increasing media reports on salt and health by involving media reporters in a randomized trial on blood pressure lowering effect of salt reduction.

Methods:

A before-and-after study with an embedded randomized controlled trial will be conducted in Changzhi, Shanxi Province of China, to evaluate whether the innovative ideas put forward in this topic (let the reporter as a researcher to observe the actual effect of the salt reduction) are effective to increase the number of media reports on salt and health in the local media over the short and long-term; and therefore to change the population's knowledge, belief and behaviours on salt intake and health among local citizens.

Research subjects:

Each reporter was responsible to recruit 4 families to participate in the trial, with two members in each family. In this research,about 60 media reporters will recruite a total of 240 families and 480 study participants.

Interventions and Main outcome measures:

Information intervention: Ask the reporter, as a researcher, to personally measure the blood pressure of the participating members and observe the effect of low sodium salt on blood pressure. At the same time, sharing the low sodium salt related knowledge of education with reporters in the form of text messages, mail and other forms. The main evaluation index is the number of reports published before and after intervention.

Low sodium salt intervention: according to the random control method, the participating families were divided into intervention group and control group. The intervention group was given free low sodium salt intervention, the control group did not have any measures, the intervention lasted for 2 months; evaluation index: blood pressure value.

Statistical methods:

Using Epidata software to establish database, input, management data, using Spss20.0 for data analysis.

1. BACKGROUND

1) Current situation of cardiovascular diseases and hypertension

Both in developed and developing countries, cardiovascular and cerebrovascular diseases have become one of the leading causes of death among residents. According to the China Cardiovascular Disease Report 2007, the estimated number of cardiovascular diseases in China is at least 230 million cases, and 3 million cases die every year from about 40% of the total causes of death of cardiovascular and cerebrovascular nationals, is the number one killer of our residents. There are many risk factors for cardiovascular and cerebrovascular diseases, among which hypertension is the most important risk factor for Chinese residents.

2) Effects of low-salt diet on blood pressure

The results of the survey on nutrition and health status of Chinese residents in 2002 showed that the higher the amount of salt, the higher the systolic and diastolic blood pressure levels of the population, compared with 6 g < daily salt intake, 12 g of daily salt intake increased the risk of hypertension by 14%, and 18 g of daily salt intake increased the risk of hypertension by 27%. Many observational studies 5,6 and randomized clinical trials4,7,8,9 have shown that reducing salt intake lowers blood pressure levels. At the same time, the decrease of blood pressure can reduce the risk of cardiovascular and cerebrovascular events 10, 11, 12. Salt is like an invisible killing Hand ", will unconsciously make people suffer from high blood pressure and other cardiovascular and cerebrovascular diseases; low-salt diet is precisely an economic, better effect of prevention of cardiovascular disease measures.

3) The role of mass media in health education

The mass media is the main source of public health information, whether at the individual or the mass level, the health information transmitted by the mass media will affect the public's perception of health. In a sense, the mass media constructs the concept of people's health in daily life, affects the way of life of ordinary people, and changes the bad habits of ordinary people.

Combined "health education" and "mass media" effectively, through the mass media to strengthen the effective dissemination of health policies and health knowledge, enhance people's awareness of health, popularize knowledge of disease prevention, develop good hygiene habits, choose the right health behavior and lifestyle, will play an important role in the prevention of disease (especially with health, living habits and other close relationship between cardiovascular and cardiovascular disease prevention)

process. Therefore, integrating health education into the mass media is a long-term, large-scale, strong impact of health education mode 14.

In the past, newspapers and radio were the main means of disseminating information to the public. Nowadays, the emergence and development of new media, such as TV, Internet, mobile phone and so on, make the influence range and strength of mass media more and more, and make effective use of mass media to carry out health education. By repeatedly propagating in the mass media, the Japanese government made people aware the harm of salt in their daily work and life, and effectively changed the bad eating habits according to the method of media propaganda. Compared with the late 1950s, the death rate of stroke in Japan decreased by 80%.

4) Role and influence of journalists in the media

The media reporter is the general name of the media personnel engaged in information collection, compilation and news reporting, is an essential part of the mass media, is the leader of the mass media, and determines the leading direction of the mass media speech. The main duty of media reporters is to be close to the masses, to life, to reality, to reveal sharp social contradictions from a keen perspective, to pay attention to profound social problems, and to record the social changes bit by bit. The reporter is not only the transmitter of the real information, the watchman of the environment, but also the enlightenment of the people's thought in a certain sense. In old times In the eyes of the surname, the voice of the reporter is the voice of the government, the behavior of the reporter is the right action, and the reporter is the embodiment of justice. Therefore, the influence of journalists' words and deeds is much higher than that of other people. If the reporter can through the mass media platform, combined with a variety of forms, long-term active and active participation in health education, the effect of health education will be far more than the general form of health education, play a multiplier effect with half the effort.

5) The significance of this study

To sum up, the popularization of public health knowledge needs the participation of mass media, and the combination of health education and mass media has become a new inevitable trend. As the main body of mass media, media journalists guide the leading direction of mass media speech, and their attitude to health education knowledge will directly affect the long-term effect of health education. Therefore, how to effectively mobilize the initiative and voluntary participation of media journalists in health education is a topic worthy of discussion. Through this research, we hope to

explore the effective ways to stimulate the enthusiasm of media journalists, and provide the basis for the sustainable and efficient development of the health education role of mass media in the future.

2. RESEARCH PURPOSES

General purpose:

The research aims to assess the effects of a novel mass media intervention in increasing media reports on salt and health by involving media reporters in a randomized trial on blood pressure lowering effect of salt reduction.

Specific objectives:

1) Increase the number of media reports on salt and health in the local media over the short and long-term by involving the reporter as a researcher to observe the actual effect of the salt reduction in lowering blood pressure among their own families, neighbours or relatives, .

2) Change the population's knowledge, belief and behaviours on salt intake and health among local citizens.

3. METHODS

Study design

We employed a before-and-after comparative study design to evaluate the effects of the intervention. Using the pre-intervention comparison as the main design, we evaluate whether the innovative ideas put forward in this topic (let the reporter as a researcher to observe the actual effect of the salt reduction test to affect the behavior and quantity of their writing and reporting related health knowledge) are effective for the intervention of journalists. The intervention to journalists is actually to invite journalists to participate and be responsible for the implementation of a rigorous randomized open-control trial to personally evaluate the effect of low sodium salt on lowering blood pressure. The invited reporter will be responsible for the subjects selected in the randomized controlled experiment, measure the effect of lowering blood pressure, and report the measurement data through the network to obtain the evaluation results of all the subjects.

Technical circuits

general route

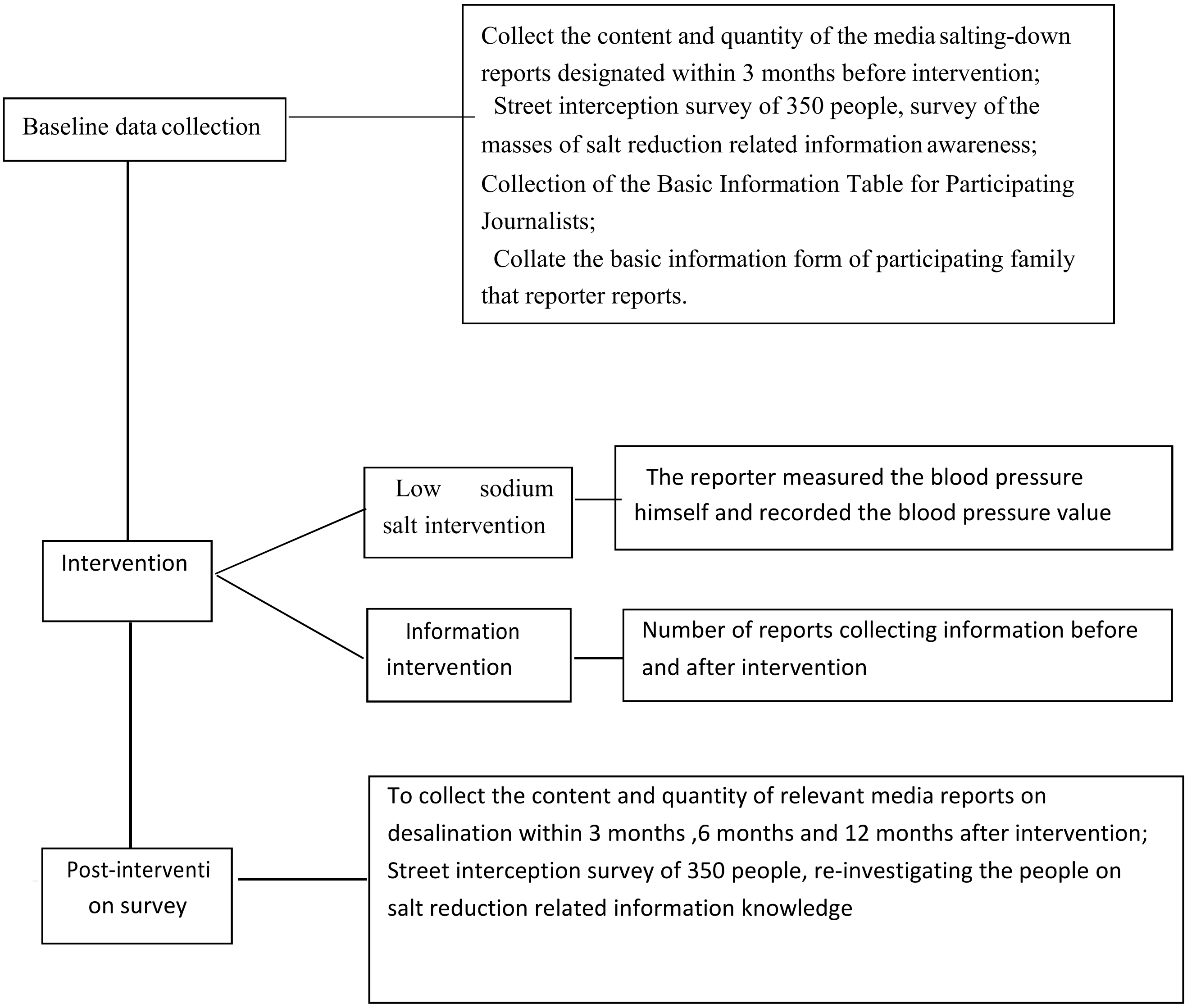

Figure 1. Overall technical circuit diagram

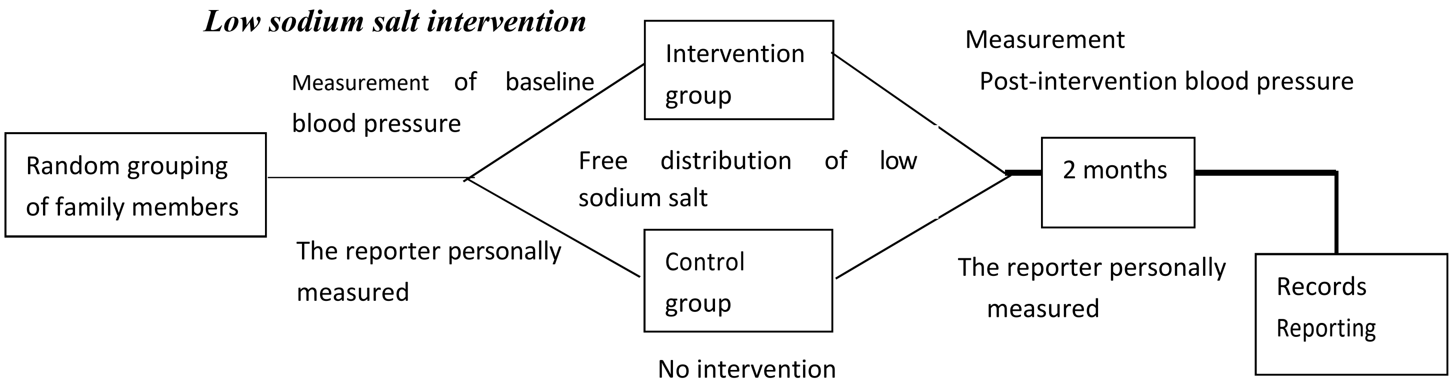

Figure 2. Low sodium salt intervention circuit diagram

Specific design*Intervention against media journalists*

1) Selection criteria for media journalists

a. Professional journalists with press cards, health columnists are preferred;

b. To reside in the Changzhi region within the next 12 months;

c. There are at least four close, accessible families of relatives or friends, and at least two ≥50 year old members of each family eat at home three meals a day;

d. Voluntary participants after informed consent.

2) Information interventions

The third month of intervention, the project team through the press conference, email, SMS and other forms, timely feedback of the results of the intervention to the participating journalists. At the same time, through the above form, a lot of knowledge about the relationship between salt reduction and health is disseminated to participating journalists.

3) Evaluation indicators

Indicator 1: Number of relevant reports on salt reduction knowledge

● Intervention objectives:

The amount of salt reduction information reported by relevant media in 3 months after intervention was 100% higher than that in 3 months before intervention.

● Information collection:

All news reports on salt-related content require collection, including:

a. The prevention and treatment of chronic non-communicable diseases, which contains salt-related elements;

b. Report on health effects of low/high salt diet;

c. Reports on the classification and characteristics of salts;

● Collection scope: Changzhi Daily (including health. Comprehensive section), Evening News (including Health Weekly)

● Collection methods:

Data are collected every 1 week by specialists using a retrospective recording method. Check the first week of "Changzhi Daily" and "Shangdang Evening News" electronic version, record the required reports;

Order Changzhi Daily and Shangdang Evening News to supplement the missing parts of the electronic version.

● Collection schedule:

Stage 1: 3 months before intervention, collect the reports that meet the requirements.

Stage 2: 3 months during intervention, collect the reports that meet the requirements.

Stage 3: 3 months after intervention, collect the reports that meet the requirements.
Stage 4: the sixth month after the intervention, collect the report that the month "Changzhi Daily" and "Shangdang Evening News" meet the requirements.
Stage 5: the 12th month after the intervention, collected the report of the relevant knowledge of Changzhi Daily and Shangdang Evening News in that month.
● Quality control: by the project site responsible personnel, the first five days of each month all related media-related information reported content and quantity.

Indicator 2: Awareness of Salt Reduction Knowledge

For the resident convenience sample surveys, with reference to previous studies [38], we assumed that the proportion of Chinese residents with the knowledge, belief and behaviour on salt intake and health in this part of China was 30%,60% and 30%. We assumed α = 0.05,so a study sample size with 400 individuals in each survey would have 85% power to detect an effect size of 10% change in each of these proportions.On the weekend before and after the intervention ,people who were passing by and willing to be investigated were investigated by means of street interception survey in front of Xiamen and August 1 Square, Changzhi City, where the flow of people was relatively large.

Table 1. List of people surveyed by street interception

Age group	Male	Female	Total
31-40 years	60	60	120
41-50 years	60	60	120
51-60 years	50	50	100
Over 60 years	30	30	60
Total	200	200	400

Interventions for participating family members

1) Selection criteria for participating family members

a. member ≥ age 50;
b. Family members who eat at home > five days a week;
c. Exclude severe kidney disease and those who are in the process of potassium diuretic therapy;
d. Able to participate in the whole project without long-term travel arrangements;
e. Voluntary participants after informed consent.

2) Calculation of sample content

As a result of this study, the blood pressure of the intervention group and the control group was compared, considering that the family was the random unit, and the analysis was carried out at the individual level, so the sample size was calculated by using the two sample mean comparison process designed randomly in the PASS11 software group:

Assuming that 2 family members per household, with an internal blood pressure correlation coefficient ICC=0.2(no intra-group correlation data between family members is currently available), a minimum of 180 families (90 in each group) are required to obtain a 90% assurance (1-β), at a standard deviation of 13.4 mmHg15 at a significant level of 0.05 to identify 5 mmHg of blood pressure inter-group differences. Considering the 10 per cent drop-out rate at the end of the study, 200 families were eventually required; each journalist was responsible for 4 families and required 50 journalists.

If the case of ICC=0.4 is considered, a minimum of 216 families (108 per group) is required, and a 10 per cent rate of missing visits is considered, resulting in 240 families and 60 journalists.

Based on the calculation results, it was preliminarily determined that 60 journalists, 240 families and about 480 family members were selected to participate in the study in the local media.

3) Random grouping method

In this study, families were grouped according to the simple random method:

Step 1: is to divide the four families chosen by each reporter into two groups according to the principle of age matching, each group of two families;

Step 2: Two families in each group were numbered 1 and 2 in the reporting order.

Step 3: Using the random number table, prepare two-digit random numbers for families in turn.

Step 4: The larger group of random numbers was marked as A(defined as the intervention group), and the smaller group was B(defined as the control group).

Examples:

Table 2: Model Table for Random Allocation of Participating Families

Journalist code	1				2				·······
Family groups	1		2		1		2		·······
Family code	1	2	1	2	1	2	1	2	·······
Random numbers	87	52	05	30	22	77	94	39	·······
Assignment	A	B	B	A	B	A	A	B	·······

4) Intervention - Low Sodium Salt Intervention

The intervention lasted 2 months and was randomly assigned to the families of the intervention group. The low sodium salt for 2 months was presented free of charge by

the project department. The low sodium salt must be consumed during the intervention; the control group did not receive any intervention.

Low sodium salt distribution takes the form of direct distribution by designated express delivery companies to the intervention group families.

5) Evaluation indicator - blood pressure

● Subjects:

Participation in family members is over the age of 50.

● Blood pressure measurement requirements:

Blood pressure measurement work required to participate in the study reporter personally completed and recorded blood pressure values;

Morning, fasting blood pressure; sit at least 5 minutes before measuring blood pressure; measure right arm blood pressure, re-test twice, take the average value; if the difference between systolic or diastolic blood pressure readings measured twice >5 mmHg, then measure again after 2 minutes apart, then take the average of 3 readings.

Sphygmomanometer by the project department unified equipment, the measurement method refers to the sphygmomanometer.

● Measurement cycle:

Blood pressure was measured twice at the beginning of the intervention, at the end of intervention.

Project managers will be in the corresponding time points, using SMS, e-mail and other forms to remind reporters for blood pressure measurement.

● Data upload:

After measuring blood pressure, the reporter should fill in "Table 4, the reporter measures the blood pressure list of the family members involved in the study ", and the blood pressure value measured on the same day through text messages, e-mail and other forms to report to the data manager.

Quality control

The quality control personnel of the project department, within the required time period, extract some family members to measure blood pressure.

Statistical analysis plan

EpiData software was used to establish database, input and manage data; and SPSS 20.0 was used for all data analysis.

1) The difference in number of relevant reports published in the media between pre- and post-intervention were analyzed and compared;

2)The changes of blood pressure of family members in intervention group, control group and intervention group before and after intervention were analyzed and compared;

3)The changes of people's cognition of salt reduction before and after the intervention were analyzed andd and compared

4.INFERENCES

(1) Preventing chronic disease: a vital investment. Geneve,World Health organization, 2005.

(2) Chinese cardiovascular disease report, 2007. Beijing, China Encyclopedia press, 2009.

(3) Wu Y, Huxley R, Li L, et al. Prevalence, awareness, treatment, and control of hypertension in China: data from the China National Nutrition and Health Survey 2002. Circulation.2008; 118(25):2679-2686.

(4) Nancy R Cook, Jeffrey A Cutler, Eva Obarzanek, et al. Long term effects of dietary sodium reduction on cardiovascular disease out comes: observational follow-up of the trials of hypertension prevention (TOHP).BMJ, 2007.

(5) Michel R Joffres, Norm RC Campbell, Braden Manns et al. Estimate of the benefits of a population-based reduction in dietary sodium additives on hypertension and its related health care costs in Canada. Can J Cardiol, 2007,23(6):437-443.

(6) INTERSALT Cooperative Research Group. INTERSALT: an international study of electrolyte excretion and blood pressure. Results for 24 hour urinary sodium and potassium excretion. BMJ. 1988; 287:319-328.

(7) Cutler J, Follman D, Elliott P, et al. An overview of randomized trials of sodium reduction and blood pressure. Hypertension. 1991; 17:I27-I33.

(8) Cutler J, Follmann D, Allender P. Randomized trials of sodium reduction: an overview. Am J Clin Nutr. 1997; 65(65):643S-651S.

(9) Whelton P, He J, Cutler J, et al. Effects of oral potassium on blood pressure meta-analysis of randomized controlled clinical trials. JAMA. 1997; 277(20): 1624-32.

(10) Blood Pressure Lowering Treatment Trialists' Collaboration. Effects of different blood pressure lowering regimens on major cardiovascular events: Second cycle of prospectively designed overviews. Lancet. 2003; 362:1527-1535.

(11) PROGRESS Collaborative Group. Randomized trial of a perindopril-based blood pressure lowering regimen among 6,105 individuals with previous stroke or transient ischemic attack. Lancet. 2001; 358:1033-1041.

(12) Bosch J, Yusuf S, Pogue J, et al. Use of ramipril in preventing stroke: double blind randomized trial. Bmj. 2002; 324(7339):699-702.

(13) http://news.ifeng.com/gundong/detail_2011_07/22/7869153_0.shtml

(14) Xu Renfa, Xu Yiwen, Ma Yu. Significance and role of mass media in health education. China health education, 2010, 26 (1): 67-69.

(15) Hu Jihong, Wu Yangfeng, Zhao LC. Randomized double-blind controlled trial of antihypertensive effect of substitute salt on patients with hypertension and their family members. Proceedings of the 9th academic annual meeting of Danone nutrition center, 2006, 144-151.

(16) Zhang J, WuT, Chu HL, et al. Salt intake belief, knowledge, and behavior: a cross-sectional study of older rural Chinese adults. Medicine (Baltimore) 2016;95:e4404.

5.ANNEX:

Annex 1

Table 1: basic information questionnaire of participating journalists Hello, I am very glad that you can participate in this research project! In order to feed back the research progress and related information to you in future work, I hope you can complete the following form for easy contact, which will not involve your privacy. Moreover, we will keep your information confidential. Please fill it in carefully. Thank you for your cooperation and support!

1. full name: _____________
2. Gender: |_ | 1 = male; 2 = female
3. Nationality: |_ | 1 = Han nationality; 2 = Hui nationality; 3 = others______
4. Date of birth: |___|___|___|___|Year|__ |Month

5.Work unit : ______________

6.Working hours: |___|___|___|___| Year

7.Contact information (required):

Mobile:___________(required) fixed telephone: _____________

Email:_________________

8. Highest education level: |__| 1 = junior high school or lower; 2 = senior high school /technical secondary school; 3 = junior college; 4 = University; 5 = graduate or above
9. The main contents of your report at this stage are as follows: | |
   1 = current political news; 2 = economy; 3 = healthy life; 4 = science and technology education; 5 = sports; 6 = entertainment; 7 = other__________
10. What sections have you been involved in|___
   1 = current political news; 2 = economy; 3 = healthy life; 4 = science and technology education; 5 = sports; 6 = entertainment; 7 = other__________

Thank you for your cooperation!

Annex 2

ID: |__|__|-|__|__|__|

Table 2. Random street crowd questionnaire

1. Essential information

1.1 Gender: |__| 1 = male; 2 = female

1.2 Date of birth: |__|__| |

1.3 Current residence:________City_______City_______District______County

1.4 Nationality: |__| 1 = Han nationality; 2 = Hui nationality; 3 = others __

1.5 Your occupation: | | 1 = workers; 2 = farmers; 3 = cadres; 4 = teachers; 5 = others_______

1.6 Highest education level: | | 1 = informal schooling; 2 = primary school; 3 = junior high school; 4 = senior high school/technical secondary school; 5 = University or above

2.Knowledge of high salt diet

2.1Do you think eating more salt is harmful to your health? | |

1 = Yes (answer 2.1.1) 2 = no (answer 2.2) 3 = don't know (answer 2.2)

2.1.1If so, what diseases can be caused or aggravated | |__|__|

1 = hypertension 2 = stroke 3 = gastric cancer 4 = memory loss 5 = other _______

2.2 What kind of salt do you eat at home? |__|

1 = iodized salt 2 = selenium salt 3 = low sodium salt 4 = coarse salt 5=hear nothing of

2.3 Do you know what the experts recommend salt intake? | _ |

1 =3g 2 = 6g 3 = 10g 4 = don't know

2.4 How did you get the above information: _______

1 = TV 2 = broadcast 3 = newspaper 4 = Internet 5 = books 6 = others_______

2.5 How much salt do you think you eat every day?

1 = Yes (please fill in)____________ 2 = don't know

Thank you for your cooperation!

Annex 3 table 3: basic information of participating families reported by the reporter

Name of reporter:______________, Affiliated unit________________, Number:☐☐☐

Family coding	Home address	Population	Contact number	Family members over 25 years old											
1				Name of head of household:		name:		name:		name:		name:		name:	
				Gender	Age	Gender	Age	Gender	Age	Gender	Age	Gender	Age	Gender	Age
2				Name of head of household:		name:		name:		name:		name:		name:	
				Gender	Age	Gender	Age	Gender	Age	Gender	Age	Gender	Age	Gender	Age
3				Name of head of household:		name:		name:		name:		name:		name:	
				Gender	Age	Gender	Age	Gender	Age	Gender	Age	Gender	Age	Gender	Age
4				Name of head of household:		name:		name:		name:		name:		name:	
				Gender	Age	Gender	Age	Gender	Age	Gender	Age	Gender	Age	Gender	Age

Annex 4 Table 4. Blood pressure measurement table of family members participated in the study

Name of reporter:______________, Affiliated unit________________, Number: ☐☐☐

Family coding	Family members over 50 years old									
	systolic pressure	Diastolic pressure	systolic pressure	diastolic pressure	systolic pressure	Diastolic pressure	systolic pressure	Diastolic pressure	systolic pressure	Diastolic pressure
1	Name of head of household:		name:		name:		name:		name:	
The first time										
The second time										
2	Name of head of household:		name:		name:		name:		name:	
The first time										
The second time										
3	Name of head of household:		name:		name:		name:		name:	
The first time										
The second time										
4	Name of head of household:		name:		name:		name:		name:	
The first time										
The second time										

Annex 5

Table 5.Collection of reports on salt reduction in relevant media

Media name	time	content	number	remarks

Annex 6

Informed consent of participating family members

(In duplicate, one copy for the investigator and one for the respondent.)

As one of the closest friends of journalists who participated in our project, you have the honor to be recommended to our project by him/her. We sincerely welcome you to join us. Please read this informed consent form carefully and make a decision on whether to participate in the study. When the researcher discusses the informed consent form with you, you can ask him/her to explain to you what you don't understand. We encourage you to have a full discussion with your family and friends before making a decision to participate in this study. The content/nature, risks, inconvenience or discomfort and other important information of this study are as follows:

The research will be carried out by the China George Institute of health led by Professor Wu Yangfeng and the Preventive Medicine Department of Changzhi Medical College led by Professor Feng Xiangxian.

Why do we do this research?

Observe the effect of low sodium salt on blood pressure and improve the bad eating habits of the participants.

Who can participate in this project?

Families close to the reporter, easy to follow-up, at least one family member aged over 50 years old. Nearly 480 people participated in this study.

What do participants do?

At the beginning of this study and at the end of two months, you need to cooperate with your reporter friends to give you free blood pressure measurement.

How long does the project last?

The study will last for 2 months, during which you are free to opt out.

What are the risks of the project?

There is no risk in this study and no loss will be caused to you. And you don't have to bear any costs.

What are the benefits of the project?

During the study, your reporter friend will take your blood pressure regularly.
If in the low sodium salt intervention group, you and your family can also get free low sodium salt for 2 months.

Will my information be kept secret?

Your personal privacy will not be involved in the research, and your personal information will be kept confidential. At the end of this study, your information collected will be deleted and destroyed, and your personal information will not appear in our research report.

Do I have the right to refuse to participate or withdraw?

It is up to you to decide whether to participate in the study. If you want to quit at any time during the study, you are free to leave without giving any reason. If you don't want to, you can refuse to provide any personal information or medical records. Your decision to refuse to participate or quit will not penalize you or lose any of the benefits you have won.

If I have any questions, who can I contact?

If you have any questions about participating in this survey, please contact the project leader in the following ways:

Huang Liping Teacher

Room B1302, Jinqiu international building, 6 Zhichun Road, Haidian District, Beijing

Beijing, China 100088

Tel: 010-82800577

E-mail：hliping@georgeinstitue.org.cn;

Li Zhifang lecturer

Changzhi Medical College, 161 Jiefang East Street, Changzhi

Changzhi, Shanxi 046000

Tel: 0355-3151455

E-mail：lzfmuzi@163.com

Statement of informed consent

"I have been informed of the contents, steps, risks and benefits of this study. I have been told who I can contact at any time when I have problems or want to get further information. I have read this informed consent and agree to participate in the study and know that I can withdraw from the study at any time; I have been told that I will receive a copy of this informed consent. "

Signature of participants____________________date_____________________

Investigator signature ____________________date_____________________
