# Supplementary material for: Randomised trial on effect of involving media reporters in salt reduction programme to increase media reports and the public’s knowledge, belief and behaviors on salt and health: Changzhi reporters trial
Source: PLoS One. 2021 Jul 20;16(7):e0252989. doi: 10.1371/journal.pone.0252989 (PMC8294101; doi:10.1371/journal.pone.0252989)
Supplement: S4 File — (PDF) [file pone.0252989.s005.pdf]

记者参与减盐研究对减盐相关知识报道的影响

参与机构及院校

中方机构	外方机构
北京大学医学部<br>长治医学院	伦敦帝国学院

研究组主要人员

项　目　负　责　人：武阳丰，北京大学医学部临床研究所
项目协同负责人：李志芳，长治医学院
外方项目负责人：**Paul Elliott**，伦敦帝国学院

研究摘要

研究目的:

探讨记者在减盐健康教育活动中的作用及提高其对大众减盐宣传报道数量、质量的方法。

研究方法：

采取嵌套式设计。采取干预前后比较作为主设计，评价本课题所提出的创新设想（让记者作为研究者观察减盐试验的实际效果来影响其撰写和报道相关健康知识的行为和数量）对记者的干预是否有效。

研究对象：

选择 60 名当地记者参加研究，每名记者推荐 4 户家庭，每户家庭最少有 2 名家庭成员参与，约有 480 名家庭成员。

干预措施及评价指标：

信息干预：让记者作为研究者，由其亲自测量参与成员血压，观察低钠盐对血压的影响。同时，以短信、邮件等形式，对记者进行低钠盐相关知识的宣教。主要评价指标是干预前、后，发表相关报道的数量；

低钠盐干预：按照随机对照方法，将参与家庭分为干预组和对照组。干预组给予免费低钠盐干预，对照组无任何措施，干预为期 2 月；评价指标：血压值。

统计方法：

采用 Epidata 软件建立数据库、录入、管理数据，采用 Spss18.0 进行数据的分析。

目　　录

一、研究背景
1. 心脑血管疾病及高血压流行现状..1
2. 低盐饮食对血压的影响..1
3. 媒体宣传在大众生活中的作用...1
4. 记者在媒体作用及影响..1
5. 本次研究的意义..2
二、研究目的..2
三、研究方法..2
1. 总体设计..2
2. 技术线路..2
3. 具体设计..3
3.1 针对参与记者的干预..3
3.1.1 媒体记者的选择标准..3
3.1.2 干预措施—信息干预...4
3.1.3 评价指标...4
3.2针对参与家庭成员的干预...5
3.2.1 家庭成员选择标准...5
3.2.2 样本含量计算..5
3.2.3 随机分组方法..5
3.2.4 干预措施..6
3.2.5 评估指标..6
3.3 质量控制..6
3.4 统计分析计划...6
四、时间进度..7
五、参考文献..8
六、附件...10
6.1 附件1 《表1、参与记者基本信息调查表》.........................10
6.2 附件2 《表3、街头随机群众调查表》...............................11
6.3 附件3 《表4、记者上报参与研究家庭基本信息表》...............12
6.4 附件4 《表5、记者测量参与研究家庭成员血压一览表》........13
6.5 附件5 《表6、相关媒体减盐相关报道收集一览表》...........14
6.6 附件6 《参与家庭成员知情同意书》...................................15

一、研究背景

1. 心脑血管疾病及高血压流行现状

无论在发达国家还是在发展中国家，心脑血管疾病都已成为居民的最主要死亡原因之一[1]。据中国心血管疾病报告 2007 报道，估计我国心血管疾病现患病人数至少 2.3 亿例，每年有 300 万例死于心脑血管国民全部死亡原因的 40%左右，是我国居民的头号杀手[2]。心脑血管疾病有众多的危险因素，其中高血压是我国居民最重要的危险因素[3]。

1.　低盐饮食对血压的影响

2002 年，我国居民营养与健康状况调查结果表明：食盐量越高，人群收缩压、舒张压水平越高，与每日食盐摄入量<6g 者相比，每日食盐摄入量≥12g 者患高血压的风险增高 14%，每日食盐摄入量≥18g 者患高血压的风险增高 27%。很多的观察性研究[5,6] 和随机临床试验[4,7,8,9]均已证实减少食盐的摄入能够降低血压水平。同时，血压的下降又能降低心脑血管事件的发病风险[10,11,12]。由此可见，盐就像“无形杀手”，会在不知不觉中使人患上高血压等心脑血管疾病；低盐饮食恰恰就是一种经济的、效果比较好的预防心血管病的措施。

3. 大众媒体在健康教育中的作用

大众传媒是公众健康信息的主要来源，无论在个人还是大众层次，大众传媒所传递的健康信息都会影响公众对健康的感知。在某种意义上说，大众传媒建构了日常生活中民众的健康观念、影响了普通老百姓的生活方式，改变了普通大大众不良的生活习惯。
若“健康教育”与“大众传媒”能有效结合起来，通过大众媒体加强卫生政策和健康知识的有效传播，增强人们对健康的认识，普及疾病预防知识，养成良好的卫生习惯，选择正确的健康行为和生活方式，将在疾病预防（尤其跟卫生、生活习惯等关系密切的心脑血管疾病预防）过程中发挥重要的作用。因此，将健康教育融入到大众传媒中的健康教育模式就是一种长期的、影响范围大、影响力度强的健康教育方式[14]。

过去，报纸，广播是主要向大众传播讯息的方式。现如今，电视、网络，手机等新媒体的出现以及发展壮大，使得大众传媒的影响范围和力度越来越大，有效地利用大众传媒进行健康教育的影响面、影响力度也就越来越大。日本政府通过反复在大众媒体上宣传，使人们在日常工作生活之余，了解到盐的危害，并根据媒体宣传的方法有效地改变不良的饮食习惯，与20世纪50年代末比较，日本脑卒中死亡率下降了80%[13]。

4. 记者在媒体作用及影响

媒体记者是媒体从事信息采集、编撰和新闻报道工作人员的通称，是大众媒体必不可少的部分，是大众传媒的主导者，决定大众传媒言论的主导方向。媒体记者的主要职责是贴近群众，贴近生活，贴近实际，以敏锐的视角揭示尖锐的社会矛盾，关注深刻的社会问题，记

录点滴的社会变迁。记者不仅是真实信息的传递者，环境的守望者，一定意义上还是民众思想的启蒙者，他们以引导舆论、激浊扬清、分析深刻、观点鲜明而受到老百姓的青睐。在老百姓的眼里，记者的声音就是政府的声音，记者的行为就是正确的行动，记者就是正义的化身。因此，记者言行的影响力度要远远高于其他人群。如果记者能够通过大众媒体的平台，结合多种多样的形式，长期的积极、主动参与到健康教育中，以身说法，那么健康教育的效果将会远远超过一般形式的健康教育，起到事半功倍的作用。

5. 本次研究的意义

综上所述，公众健康知识的普及需要大众传媒的参与，健康教育与大众传媒的结合已成为新的必然趋势。而媒体记者作为大众传媒的主体，引导大众媒体言论的主导方向，其对健教知识的的态度也将会直接影响到健康教育的长期效应。因此，怎样有效地调动媒体记者主动自愿参与健康教育的积极性是一个值得探讨的课题。通过本次研究希望能够探讨激发媒体记者积极性的有效方法，为以后能够持续、高效发挥大众传媒的健康教育作用提供依据。

二、研究目的

总目的：

探讨记者在减盐健康教育活动中的作用及提高其对大众减盐宣传报道数量、质量的方法。

具体目的：

1. 通过让媒体记者亲自参与对研究对象的选择、分组、血压测量等研究工作，观察低盐饮食降低血压的作用。
2. 增加媒体记者对低盐饮食降低血压的信心，激发他们自觉自愿的加入到宣传低盐饮食的队伍中来，以发挥他们的积极作用。

三、研究方法

1．总体设计：

这是一个嵌套式设计。采取干预前后比较作为主设计，评价本课题所提出的创新设想（让记者作为研究者观察减盐试验的实际效果来影响其撰写和报道相关健康知识的行为和数量）对记者的干预是否有效。对记者的干预实际上是邀请记者参与并负责实施一项严格的随机开放对照试验，亲自评价低钠盐降低血压的效果。受邀记者将负责入选随机对照实验的研究对象，测量降压效果，并通过网络上报测量数据，获得全体研究对象的评价结果。

2. 技术线路：

2.1 总体线路

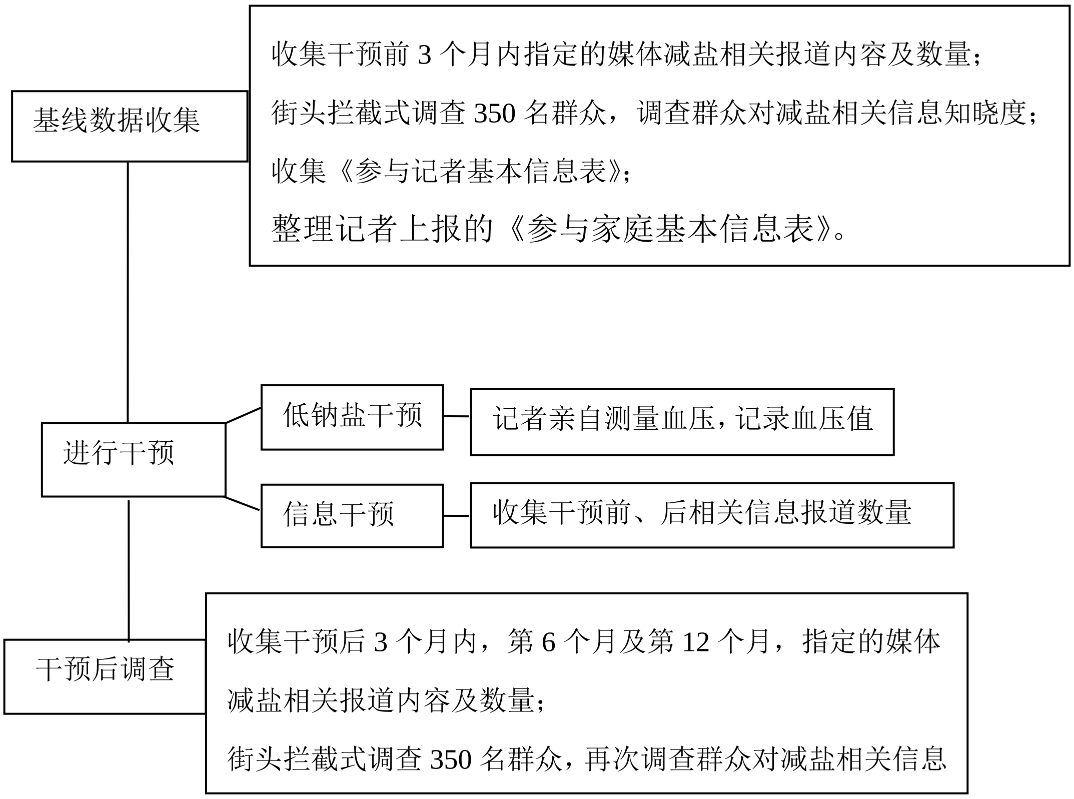

图 1、总体技术线路图

2.2 低钠盐干预

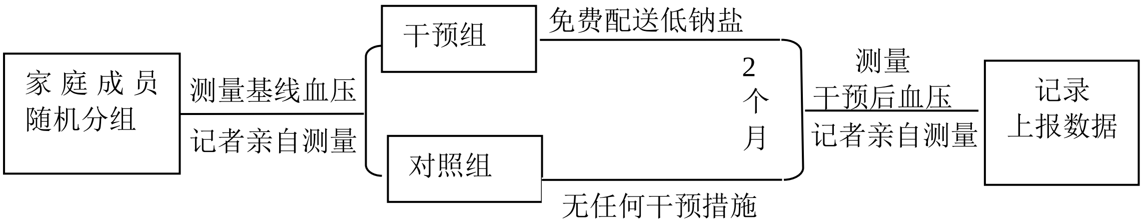

图 2、低钠盐干预线路图

3.具体设计

3.1 针对媒体记者的干预

3.1.1 媒体记者的选择标准

① 持有记者证的专业记者，健康专栏记者优先纳入；

② 未来 12 个月内居住在长治地区；

③ 有至少 4 个关系亲密的、能够方便到达的亲戚或朋友家庭，而且每个家庭中至少有 2 位≥50 岁成员每日三餐在家就餐；

④ 知情同意后志愿参与者。

3.1.2 干预措施----信息干预

干预第 3 个月，项目组通过记者会、电子邮件、手机短信等形式，及时将干预结果反馈给参与记者。同时，通过以上形式，将大量关于减盐与健康关系的知识传播给参与记者。

3.1.3 评价指标

指标一、减盐知识相关报道数量

● 干预目标：

干预后 3 个月相关媒体报道减盐信息量较干预前 3 个月提高 100%。

● 收集信息内容：

凡与盐有关内容的新闻报道均要求收集，主要包括：

① 涉及慢性非传染性疾病防治内容，且其中含有与盐有关的内容；

② 涉及低盐/高盐饮食对健康影响的报道；

③ 涉及盐的分类及特点的报道；

● 收集范围：长治日报（含健康.综合版块）、上党晚报（含健康周刊）

● 收集方法：

采用回顾式记录方法，由专门人员每隔 1 周收集一次数据。

查阅前 1 周《长治日报》和《上党晚报》电子版，记录符合要求的报道；

订购《长治日报》和《上党晚报》，对电子版中有缺失部分加以补充。

● 收集时间安排：

第一阶段：干预前 3 个月，收集符合要求的报道。

第二阶段：干预时 3 个月，收集符合要求的报道。

第三阶段：干预结束后 3 个月，收集符合要求的报道。

第四阶段：干预后第 6 个月，收集当月《长治日报》和《上党晚报》符合要求的报道。

第五阶段：干预后第 12 个月，收集当月《长治日报》和《上党晚报》相关知识的报道。

● 质量控制：　由项目现场负责人员,每月前五日时间段内所有涉及媒体相关信息报道内容及数量。

指标二、民众减盐知识知晓率

干预前 1 周末和干预后 1 周末，在长治市人流量比较大的长治市金威商厦门前、长治八一广场，由经过统一培训的人员，采用街头拦截式调查方法，对路过且愿意接受调查的群众进行《表 2 街头随机群众调查表》。参考以往的研究[17]，假设对食盐摄入和健康有知识、信念和行为的比例分别为 30%、60%和 30%。以 α = 0.05 为检验水准，每个方面变化 10%，达到 85%的检验效能，需 400 例研究对象。

表 1、街头拦截式被调查群众明细表

年龄组	男性	女性	合计
31-40 岁	60	60	120
41-50 岁	60	60	120
51-60 岁	50	50	100
60 岁以上	30	30	60
合计	200	200	400

3.2 针对参与家庭成员的干预

3.2.1 参与家庭成员的选择标准

① 年龄≥50 岁成员；

② 每周在家里就餐>5 天的家庭成员 ；

③ 排除严重的肾脏疾患，及正在做保钾利尿治疗者；

④ 能够全程参加项目，没有长期外出安排者；

⑤ 知情同意后志愿参与者。

3.2.2 样本含量的计算

本研究结果是干预组和对照组的血压比较，考虑是以家庭为随机单位，而进行的是个体水平的分析，故用 PASS11软件中组群随机设计的两样本均数比较过程来计算所需样本量：

假定每户2名家庭成员，家庭成员内部血压相关系数 ICC=0.2（目前未找到家庭成员之间的组内相关性资料），则最低需要180个家庭（每组90个），才可以在0.05的显著性水准 α 下获得90%的把握度（1-β），在标准差为13.4mmHg[15]时，得以识别5mmHg 的血压组间差异。考虑研究终点时10%的家庭失访率，则最终需要200个家庭；每名记者负责4户家庭，需要记者50名。

如果考虑 ICC=0.4的情况，则最低需要216个家庭（每组108个），考虑10%的失访率，则最终需要240个家庭，记者60名。

根据计算结果，初步确定在当地媒体选择60名记者，240个家庭，约480名家庭成员参加本次研究。

3.2.3 随机分组方法

本研究参与家庭分组按单纯随机法进行分组:

第一步：将每个记者选择的四个家庭，根据年龄匹配原则分为两组，每组两个家庭；

第二步：每组中的两个家庭按上报顺序编号为1和2。

第三步：利用随机数字表，依次给家庭编制两位的随机数字。

第四步：随机数字较大一组的标记为 A（规定为干预组），较小的为 B（规定为对照组）。

举例：　　　　表 2、参与家庭随机分配示范表

记者编码	1				2				…….
家庭分组	1		2		1		2		
家庭编码	1	2	1	2	1	2	1	2	…….
随机数字	87	52	05	30	22	77	94	39	…….
赋　值	A	B	B	A	B	A	A	B	…….

3.2.4 干预措施-----低钠盐干预

干预为期 2 个月，随机分配到干预组的家庭，由项目部免费赠送为期 2 个月的低钠盐。干预期间必须食用所配送的低钠盐；对照组不接受任何干预。

低钠盐配送采取由指定快递公司直接配送到干预组家庭的方式。

3.2.5 评价指标----血压

● 测量对象：

参与家庭中年龄大于 50 周岁的家庭成员。

● 血压测量要求：**血压测量工作要求参与研究记者亲自完成，并记录血压值；**

晨起、空腹血压；测量血压前最少静坐 5 分钟；测坐位右臂血压、重复测两次，取平均值；如果 2 次测量的收缩压或舒张压读数相差>5mmHg，则相隔 2 分钟后再次测量，然后取 3 次读数的平均值。

血压计由项目部统一配备，测量方法参考血压计使用说明书。

● 测量周期：

在干预开始、第 2 月末，测量两次血压。

项目分管人员会在相应时间点，采用短信、电子邮件等形式提醒记者进行血压测定。

● 数据的上传：

记者测定血压后，需填写《表 4、记者测量参与研究家庭成员血压一览表》，且在当日将测量的血压值通过短信、电子邮件等形式上报给数据管理人员。

3.3 质量控制：

由项目部质控人员，在要求时间段内，抽取部分参与家庭成员测量血压值。

3.4 统计分析计划：

采用 Epidata 软件建立数据库、录入、管理数据，采用 Spss18.0 进行数据的分析。

3.4.1　分析比较干预前、后，发表在媒体相关报道数目的变化；

3.4.2　分析比较干预组和对照组及干预组干预前、后参与家庭成员血压的变化；

3.4.3　分析比较干预前后，民众对减盐知识认知的改变；

四、参考文献

1.Preventing chronic disease:a vital investment.Geneve,World Health organization,2005.

2.卫生部心血管病防治中心.中国心血管病报告2007.北京，中国大百科全书出版社，2009

3.Wu Y, Huxley R, Li L, et al. Prevalence, awareness, treatment, and control of hypertension in China: data from the China National Nutrition and Health Survey 2002. Circulation.2008;118(25):2679-2686.

4.Nancy R Cook,Jeffrey A Cutler,Eva Obarzanek,et al. Long term effects of dietary sodium reduction on cardiovascular disease out comes : observational follow-up of the trials of hypertension prevention (TOHP).BMJ, 2007

5.Michel R Joffres, Norm RC Campbell, Braden Manns et al.Estimate of the benefits of a population-based reduction in dietary sodium additives on hypertension and its related health care costs in Canada.Can J Cardiol, 2007,23(6):437-443.

6.INTERSALT Cooperative Research Group. INTERSALT: an international study of electrolyte excretion and blood pressure. Results for 24 hour urinary sodium and potassium excretion. BMJ. 1988;287:319-328.

7.Cutler J, Follman D, Elliott P, et al. An overview of randomized trials of sodium reduction and blood pressure. Hypertension. 1991;17:I27-I33.

8.Cutler J, Follmann D, Allender P. Randomized trials of sodium reduction: an overview. Am J Clin Nutr. 1997;65(65):643S-651S.

9.Whelton P, He J, Cutler J, et al.Effects of oral potassium on blood pressure meta-analysis of randomized controlled clinical trials.JAMA.1997;277(20):1624-32.

10.Blood Pressure Lowering Treatment Trialists’ Collaboration. Effects of different blood pressure lowering regimens on major cardiovascular events: Second cycle of prospectively designed overviews. Lancet. 2003;362:1527-1535.

11.PROGRESS Collaborative Group. Randomized trial of a perindopril-based blood pressure lowering regimen among 6,105 individuals with previous stroke or transient ischemic attack. Lancet. 2001;358:1033-1041.

12.Bosch J, Yusuf S, Pogue J, et al. Use of ramipril in preventing stroke: double blind randomized trial. Bmj. 2002;324(7339):699-702.

13. http://news.ifeng.com/gundong/detail_2011_07/22/7869153_0.shtml

14. 徐仁发, 徐毅雯, 马昱. 大众传媒应用于健康教育的意义与作用. 中国健康教育,2010,26(1):67-69

15. 胡继红,武阳丰,赵连成.代用盐对高血压患者及其家庭成员降压效果的随机双盲对照试验.达能营养中心第九次学术年会论文集，2006,144-151

五、附件：

5.1 附件 1

表 1、 参与记者基本信息调查表

您好，非常高兴您能参加本研究项目！为了在今后工作中，将本研究进展及相关信息时反馈给您，希望您能完成以下表格，方便联系，其中不会涉及到您的隐私。而且，我们会对您的信息绝对保密,请您认真填写，感谢您的配合和支持！

1. 姓名：________________

2. 性别：　|__|　1=男；2=女

3. 民族：|__|　1=汉族；2=回族；3=其他________

4. 出生年月：|__|__|__|__|年|__|__|月

5. 工作单位：______________________

6. 参加工作时间：|__|__|__|__|年

7. 联系方式（必填）：

手机：_______________（必填）固定电话：_________________ 邮箱：_________________

8. 最高文化程度：|__|　1=初中或更低；　2=高中/中专；3=大专；　4=大学；　5=研究生及以上

9. 您现阶段参与报道内容主要是：　|__|

1=时政新闻；　2=经济；　3=健康生活　4=科技教育；5=体育；　6=娱乐；　7=其它：______

10. 你曾经参与报道过那些版块内容|__|__|__|__|

1=时政新闻；　2=经济；　3=健康生活　4=科技教育；5=体育；　6=娱乐；　7=其它：______

此次调查结束，谢谢您的合作！

5.2 附件 2

ID：|__|__|-|__|__|__|

表 2、街头随机群众调查表

1. 基本信息

1.1 性别：　|__|　1=男；2=女
1.2 出生年月：　|__|__|

1.3 现居住地：_______市______市_____区_____县

1.4 民族：|__| 1=汉族；　2=回族；　3=其他______

1.5 您的职业：　|__|　1=工人；　2=农民；　3=干部；4=教师；5=其他______

1.6 最高文化程度：　|__|　1=非正式上过学；　2=小学；　3=初中；　4=高中/中专；5=大学及以上

2. 高盐饮食相关知识

2.1 您觉得吃盐多对身体有害吗？|__|

1=有（回答 2.1.1）　2=没有（回答 2.2）　3=不知道（回答 2.2）

2.1.1 如果有，可以引起或加重哪些疾病？|__ |__|__|__|__ |__|__ |__|__ |

1=高血压　2=脑卒中　3=胃癌　4=记忆力下降　5=其他_________
2.2 您家里吃的是哪种盐？|__ |

1=加碘盐　2=加硒盐　3=低钠盐　4=粗粒盐　5=不知道

2.3 您知道专家推荐食盐摄入量是多少吗？|__ |

1=3g　2=6g　3=10g　4=不知道

2.4 您是如何获得以上信息的：______

1=电视　2=广播　3 =报纸　4=网络　5=书籍　6=其他__________

2.5 您认为自己每天大约吃进去多少盐？

1=知道（请填写）___________　2=不知道

此次调查结束，谢谢您的合作！

5.3　附件 3　　表 3、记者上报参与研究家庭基本信息表

记者姓名：____________，所属单位：___________，编号：□□□

家庭编码	家庭住址	人口数	联系电话	25 周岁以上家庭成员信息											
1				户主姓名：______		姓名：______		姓名：______		姓名：______		姓名：______		姓名：______	
				性别	年龄	性别	年龄	性别	年龄	性别	年龄	性别	年龄	性别	年龄
2				户主姓名：______		姓名：______		姓名：______		姓名：______		姓名：______		姓名：______	
				性别	年龄	性别	年龄	性别	年龄	性别	年龄	性别	年龄	性别	年龄
3				户主姓名：______		姓名：______		姓名：______		姓名：______		姓名：______		姓名：______	
				性别	年龄	性别	年龄	性别	年龄	性别	年龄	性别	年龄	性别	年龄
4				户主姓名：______		姓名：______		姓名：______		姓名：______		姓名：______		姓名：______	
				性别	年龄	性别	年龄	性别	年龄	性别	年龄	性别	年龄	性别	年龄

5.4　附件 4　　　表 4、记者测量参与研究家庭成员血压测量表

记者姓名：__________，所属单位：__________，编号：□□□

家庭编码	50 周岁以上家庭成员信息									
	收缩压	舒张压	收缩压	舒张压	收缩压	舒张压	收缩压	舒张压	收缩压	舒张压
1	户主姓名：__________		姓名：__________		姓名：__________		姓名：__________		姓名：__________	
第一次										
第二次										
2	户主姓名：__________		姓名：__________		姓名：__________		姓名：__________		姓名：__________	
第一次										
第二次										
3	户主姓名：__________		姓名：__________		姓名：__________		姓名：__________		姓名：__________	
第一次										
第二次										
4	户主姓名：__________		姓名：__________		姓名：__________		姓名：__________		姓名：__________	
第一次										
第二次										

5.5 附件 5

表 5、相关媒体减盐相关报道收集一览表

媒体名称	时间	内容	数量	备注

5.6 附件 6

参与家庭成员知情同意书

（一式二份，调查员保留一份，被调查者保留一份)

作为参与我们项目的记者朋友最亲密的人之一，您有幸被他/她推荐到我们的项目中，真诚的欢迎您的加入。请仔细阅读本知情同意书并慎重做出是否参加研究的决定。当研究人员和您讨论知情同意书的时候，您可以让他/她给您解释您看不明白的地方。我们鼓励您在做出参与此项研究的决定之前，和您的家人及朋友进行充分讨论。本研究的内容/性质、风险、不便或不适及其他重要信息如下：

武阳丰教授所领导的中国乔治健康研究所和冯向先教授领导的长治医学院预防医学教研室将开展这项研究。

为什么进行这项研究？

观察低钠盐对血压的影响，改善参与人群不良的饮食习惯。

谁可以参加此项目？

记者身边亲密的、方便随访、有年龄≧50 岁家庭成员的家庭。将近？人参与到此项研究中。

参加人员要做什么事情？

您需要在本研究开始、2 月末时，配合您的记者朋友，给您进行免费的血压测量。

该项目持续多长时间？

此项研究将持续 2 个月，期间您可以自由选择退出。

该项目有何风险？

本研究没有任何风险，不会对您造成任何损失。而且您不需要承担任何的费用。

该项目有何益处？

研究期间，您的记者朋友会定期的给您测量血压。

如果是低钠盐干预组，您及家人还可以获得为期 2 个月免费的低钠盐。

我的信息会被保密吗？

研究中不会涉及您的个人隐私，您的个人信息将得到保密。在此次研究结束后，所收集到您的信息将删除与销毁，并且我们的研究报告中也不会出现您的个人信息。

我是否有拒绝参与或者半途退出的权力？

是否参与研究完全有您决定。如果您在研究期间任何时候想中途退出，您都可以自由离开并不用说明任何理由。如果您不愿意，您可以拒绝提供任何私人信息或者医疗记录。您拒绝参与或者退出的决定将不会使您受到惩罚或者损失任何赢得的利益。

如果有问题，可以联系谁?

如果您对参加此次调查有任何问题，请按以下方式地方联系项目负责人：

黄丽平　老师

北京海淀区知春路 6 号锦秋国际大厦 B1302 室

中国　北京　100088

电话：010-82800577

E-mail：hliping@georgeinstitue.org.cn;

李志芳　讲师

长治市解放东街 161 号长治医学院

山西　长治　046000

电话：0355-3151455

E-mail：lzfmuzi@163.com

知情同意书申明

“我已被告知次研究研究内容、步骤、风险及获益情况。我已被告知当遇到问题，或想进一步获得信息时可随时与谁联系。我已阅读了此份知情同意书并同意参加这项研究，且知道自己随时可以退出本研究；我已被告知我将得到这份知情同意书的副本。”

参与人员签名________________________　　　日期__________________

调查员签名 ________________________　　　日期__________________
